# Begging Parsons

**Published:** 1887-07-09

**Authors:** 


### BEGGING PARSONS.

A CLERGYMAN in a poor London parish is almost bound to be either an angel or a devil. By a devil we do not mean a personage of splendid and heroic wickedness like Milton's Satan, or a base and vulgar fiend of the penny-dreadful type, but a blind and inconsequent idler, who seeing that men and women by the thousand are falling into moral decay and ruin, calmly folds his hands or smokes his pipe, and closes the argument with a *non possumus.* He sees around him on all hands so much helplessness; so much carelessness and indifference as the gradual result of the powerlessness; so much wickedness and cruelty on the part of the strong; so much distress, pain, and utter hopelessness on the part of the weak, that he soon recognises the necessity of either bracing himself up like a soldier and a giant to fight the evil, or of shutting his eyes tight, taking his stipend, such as it is, and "letting things drift." To the honour of human nature be it said, he very seldom takes the course of letting things drift. Whatever be his theological standpoint, whether high, low, broad, or nonconformist, the manlier and nobler part of his nature is aroused by the debased and debasing anarchy, and he metaphorically takes off his coat, tucks up his sleeves, and begins to do what the Americans call "his level best" to bring a little order out of the ruinous and heart-breaking chaos.

It is profoundly instructive to note how half-a-dozen years at the East-end will turn "a vague, wavering capability" of a curate into a prompt, energetic, and efficient man of business. The same period spent in a fashionable suburb or watering-place might have had a completely opposite effect, and made him into a poor, dawdling dispenser of pointless pulpit trifles, and of perhaps elegant, but certainly inane afternoon tea small jokes and parish gossip. Most of the ministers of religion in poor neighbourhoods are bound by the compelling influences of their environment to be honest and earnest men. That they are such there is not the least doubt; and anything they have to say about their own particular work: any appeal they feel it their duty to make for help in that work, ought at once to receive the serious attention of good and reasonable people everywhere.

As a class the clergy hate begging. All Englishmen hate begging, either for themselves or for anybody else. We would a thousand times rather work, or if the age permitted, take a sword into our hands and fight for what we want, than go cap in hand to all and sundry. And yet the clergy are most persistent beggars. It is to their honour that they are so. They see the dreadful wants and miseries which press on all sides of them; they know that some at any rate of these miseries could be mitigated or removed by money; they have not themselves a tenth part, even if they gave up all their incomes, of that which is required; and so, as we said before, to their everlasting honour be it spoken, they will consent to beg rather than see their people suffer without hope or help.

A good many appeals have come to us from different ministers of religion, and some of them tell us that their letters in the newspapers are bringing them next to nothing. What is the reason? All the money cannot have been spent on the Jubilee, on the Imperial Institute, on the hospitals—where has it gone? We know how much has been given to these various objects, and all the sums combined are a mere trifle even for London itself to give. It is the old story—people do not think. But people must think, and if they have not hitherto practised the habit they must begin to learn. This age demands thought of everybody, and will have it. Our daily business has to be grappled with manfully and handled with all our best powers. It must be the same with our charities. We admit the claims of charity generally, and we give something now and again, as we think we can afford it; but that is not the way to do. We must give our judgment to the matter, and become wise almoners of our own benevolence. That is where people fail, and that is how it happens that many institutions are prosperous which ought to have their doors closed and their windows nailed up, whilst many other good and pressing objects, like the "Children's Day in the Country" fund, are starved, crushed, and rendered powerless for want of means. There is enough money and to spare for every good object if it were all given with intelligence and discrimination.

Take these children's "day in the country" schemes in the various centres. What a very little is needed to make them all entirely successful! A few thousands of pounds given in this direction would ensure all the children of all the London slums the most complete happiness for a brief period. But when are the thousands to be given—after the summer is over, when they are no longer of any use? One clergyman writes to say that as the result of his appeal in the various newspapers he has received a certain sum. How much will our readers guess? Two shillings!!! The *Times* and the *Telegraph*, the *Standard* and the *Daily News*, the Lords and the Commons, the lawyers and the doctors, the country parsons and the squires, all these liberal and enlightened papers, and all their liberal and enlightened readers, have sent *two shillings* to a London clergyman to enable him to give nearly a thousand poor children a day in the country. After that—what? The deluge surely; but a deluge of cheques, bank-notes, guineas, half-crowns, sixpences, and even pence.

We do not blame our people for stinginess: they are not stingy: they are generous. And we know the times are hard; but there are tens of thousands of pounds given, and given carelessly, without knowledge and without judgment That is where the fault lies. These children's schemes do not require five hundred pounds, or such large sums from any one man. They are not like hospitals, huge leviathans that can never have too much. They are small affairs of a temporary character, and a few shillings from each generous heart will be abundantly enough. We do not like, where there are so many appeals, to name one or two more particularly than others, but the daily papers are full of them, and most of our readers probably know of some clergyman or minister whom they would especially like to help. In any case the summer is advancing, the town will soon be empty of its wealth, leaving its poverty behind, and it is imperative that what is going to be given be given without delay. *Bis dat qui cito dat.*

